# Editorial Perspective: Expanding the search for environmental exposures related to children's development

**DOI:** 10.1111/jcpp.70212

**Published:** 2026-08-07

**Authors:** Mark Wade, Maya Mammon

**Affiliations:** ^1^ Department of Applied Psychology and Human Development University of Toronto Toronto ON Canada

**Keywords:** Environmental exposures, environmental influences, child development

## Abstract

In the fields of child psychology and psychiatry, countless environmental factors have been identified as correlates of children's health and development, yet each of these generally accounts for a small proportion of variance. The unaccounted‐for variation in a model may be due to measurement error, random variation, model misspecifications or unmeasured predictors. The search for predictors traditionally viewed as being quite distal in children's social ecology has rapidly expanded over the past decade. For example, concerns regarding the impact of climate change on planetary health have been accompanied by research directly assessing the impact of climate‐related environmental stressors on the health and well‐being of children and youth. In this space, factors such as ambient heat exposure and air pollution during sensitive windows of development have recently been incorporated into studies examining children's mental health and neurodevelopment. In this perspective paper, we discuss the results of two such papers published in the journal, one each focused on heat exposure (Cuartas et al., 2026) and air pollution (Oh & Song, 2026) in relation to children's neurodevelopmental outcomes. Drawing on large international samples and using diverse methodologies, these studies show several small but consistent associations with a range of child outcomes, thus providing compelling initial evidence of the effects of these climate‐related environmental stressors on development. Still, many unanswered questions remain related to the generalizability and reliability of findings, the domains of development most impacted, and the specific factors that may either exacerbate or mitigate the effect of these stressors. With considerable practice and policy implications, we argue for continued work in this area using diverse samples, robust measurement tools, and integration of developmental theories that will move beyond simple associations to help capture the complex mechanisms underlying these associations.

The past several decades of research on child development have revealed a range of factors that are reliably associated with children's outcomes. These include genetic and epigenetic influences, individual differences in brain structure and function, and aspects of the home and school environment, such as family and peer relationships, early‐life adversity, the conditions of neighbourhoods, and the quality of early learning environments. Research on *sensitive periods* in development underscores how experiences during specific windows of development may produce lasting behavioural changes due to the sensitivity of specific neural circuits to input during those periods (Nelson, Sullivan, & Valdes, 2025). Similarly, frameworks such as the developmental origins of health and disease (DoHAD) model highlight the disproportionate impact of environmental exposures on development when those exposures occur during particular periods, such as gestation (Monk & Fernández, 2022). This includes poor maternal nutrition, maternal distress (e.g. depression and anxiety), and variation in maternal gestational biology (e.g. endocrine and inflammatory conditions). While most research applying a DoHAD perspective has focused on health outcomes such as diabetes, obesity, and cardiovascular disease (Tan, Zhang, Yan, & Xu, 2024), this framework has been extended to the field of psychiatry, with recent research showing that prenatal maternal mental health symptoms are associated with children's behavioural and emotional problems from mid‐childhood through adolescence, independent of genetic risk (Chen et al., 2024).

Together, this wide body of research from different fields has clearly demonstrated the impact of time‐specific environmental exposures on children's health and development. Yet in each of these studies, the proportion of variance explained in any given outcome is generally small. Even socioeconomic status—a circumstance encapsulating a host of deleterious exposures—explains only about 5% of the variance in outcomes during childhood and adolescence (Paulich, Ross, Lessem, & Hewitt, 2021; Peverill et al., 2021). While non‐trivial and potentially of great consequence, the search for additional exposures that may explain children's outcomes remains an urgent priority in the field of developmental psychopathology.

One such set of exposures that is gaining attention in the fields of child psychology and psychiatry is climate‐related environmental stressors. These include exposures that are worsened by or implicated in climate change, including heat, air pollution, noise, and chemical exposures. In particular, research on ambient heat and air quality hazards has been rising, with research underscoring the importance of both prenatal and early‐life exposure for maternal and child health and well‐being (Lei, Li, Guo, Chen, & Yi, 2025; Persson et al., 2025). While by no means a panacea to account for the considerable amount of unexplained variance in child outcomes mentioned above, this previously understudied area represents a promising direction for developmental research with both practice and policy implications.

To that end, two recent studies published in *JCPP* on climate‐related environmental stressors are worth highlighting. First, Cuartas, Balza, Camacho, and Gómez‐Parra (2026) examined the impact of ambient heat exposure on core developmental skills in nearly 20,000 children from 6 countries using geolocation and temperature data from the ERA5‐Land Monthly Aggregated Climate Dataset. They found that children aged 3–4 years exposed to average monthly maximum temperatures >32°C (relative to <26°C) were at a greater risk for experiencing parent‐reported developmental challenges, especially with literacy‐numeracy and socioemotional skills. These effects were most pronounced under conditions of social and material deprivation, suggesting a potentiation of risk in the context of both climate‐related stress and social adversity. While not an original focus of the study, there was also suggestive evidence for increased developmental risk as a function of *prenatal* exposure to higher average temperatures, though these effects were somewhat inconsistent. These results provide strong preliminary evidence for the effects of heat exposure on children's outcomes, and lay the groundwork for future generalizability studies that can test similar effects in other countries and populations. Indeed, one potential limitation of the study by Cuartas and colleagues is that the countries included (Georgia, The Gambia, Madagascar, Malawi, Sierra Leone and the State of Palestine) have high average monthly temperatures overall. For regions without such high average temperatures, alternative heat metrics—such as the presence of extreme heat events, heat waves, and temperature variability (e.g. fluctuations from expected conditions)—may be important sources of variation (Lin et al., 2025; Thompson et al., 2023). Moreover, studies that include both parent‐report and direct performance‐based assessments of child behaviour are important for future research given that, for certain outcomes (e.g. executive functioning), parent reports and direct assessments measure distinct constructs (Toplak, West, & Stanovich, 2013), and associations between particular adversity dimensions and outcomes can vary significantly based on the measurement approach (Andrews, Atkinson, Harris, & Gonzalez, 2021). Finally, research that includes a wider range of developmental outcomes will improve our understanding of the domains most strongly associated with heat exposure. This will open new avenues for future mechanistic work that explores the neurobiological, physiological, and social factors that account for these effects and the conditions under which such effects are most robust.

The second study we review is a nationwide population‐based cohort study of nearly 1.5 million children in South Korea that examined associations between prenatal exposure to multiple air pollutants (nitrogen dioxide [NO_2_], sulfur dioxide [SO_2_], carbon monoxide [CO], ozone [O_3_] and particulate matter ≤10 μm [PM_10_]) and children's risk of neurodevelopmental disorders, the latter of which was defined using International Classification of Disease (ICD) codes from the National Health Insurance Service of Korea (Oh & Song, 2026). The neurodevelopmental conditions included were intellectual disabilities, speech, language or motor delay, and common behavioural or emotional disorders (e.g. ADHD, conduct, anxiety). It was shown that maternal exposure to several air pollutants during pregnancy was related to increased risk of neurodevelopmental disorders in children, though most effects were very small. The largest effect involved NO_2_, where each 0.01‐ppm increase was associated with an 18% higher risk of having any neurodevelopmental disorder. In a more granular analysis, NO_2_ exposure was most strongly related to ADHD diagnoses compared to other neurodevelopmental disorders. SO_2_ exposure also showed some small focal associations with neurodevelopmental risk, most clearly with risk of autism spectrum disorder (ASD), and less strongly with ADHD and developmental language disorder. Third trimester‐specific NO_2_ exposure showed the strongest associations with outcomes, though effects were significant across all three trimesters. SO_2_ exposure was most significant during the first and third trimesters, but effects were small overall. Finally, sex‐stratified analysis revealed somewhat stronger effects of prenatal NO_2_ exposure in girls compared to boys, while SO_2_ showed small but significant associations in both sexes.

The study by Oh and Song (2026) represents one of the largest to date on the association between prenatal air pollution exposure and children's neurodevelopmental outcomes, using nationally standardized protocols to measure air pollution and administrative record data for children's outcomes. However, as researchers only measured air pollution exposure prenatally, it is unclear how these effects compare to exposures during early childhood. For instance, while *prenatal* PM_10_ exposure was not a significant predictor of neurodevelopmental risk, findings from recent meta‐analyses suggest that early *postnatal* exposure to this pollutant is indeed associated with increased risk of ASD (Tartaglione, Camoni, Calamandrei, Chiarotti, & Venerosi, 2024). This underscores the importance of measuring and distinguishing between timing‐based exposures to different pollutants. Another major omission from the study by Oh and Song (2026) was the lack of measurement of PM_2.5_, as nationwide monitoring of this pollutant was not implemented until after the study period. However, the neurotoxic effects of fine particulate matter such as PM_2.5_ have been identified as potential mechanisms underpinning cognitive, emotional, and behavioural difficulties (Kefas, Roy, & D'Angiulli, 2025), and studies including both NO_2_ and PM_2.5_ together have shown comparable effects on psychiatric outcomes in youth (Roberts et al., 2019). Determining the shared and unique mechanisms linking different pollutants to child outcomes remains a research priority. Such mechanisms may include neurobehavioral pathways involving oxidative stress, neuroinflammation, and maternal immune activation which may, in turn, disrupt fetal brain development, including alterations in neurogenesis, synaptogenesis, and myelination (Kefas et al., 2025). Some research points to associations between exposure to air pollution during pregnancy or early childhood with measures of brain morphology, as well as structural and functional connectivity (Guxens et al., 2022). This research again suggests potentially distinct neurobehavioural mechanisms linking prenatal versus postnatal air pollution exposure to child outcomes, though presently this remains quite speculative due to the lack of within‐participant longitudinal studies in this space.

Together, the findings of Cuartas, Balza, Camacho, and Gómez‐Parra (2026) and Oh and Song (2026) extend what is known about the effects of climate‐related environmental stressors on children's development. Beyond explaining individual variation in certain outcomes, such exposures may help to explain how broad social determinants shape population health and underlie systemic health inequities. For example, known socioeconomic and demographic disparities in mental health may be partly explained by the social stratification of air pollution exposure, with economically, ethnically, or racially marginalized groups being exposed to significantly higher levels of pollution (Jbaily et al., 2022; Woo et al., 2019). This is of substantial concern considering that ambient PM_2.5_ alone is the fifth‐ranked risk factor for global mortality, accounting for 4.2 million deaths (representing 7.6% of all global deaths) (Cohen et al., 2017). Such effects may extend to the field of psychiatry, with recent research in adults showing disparities between racial groups in mental health due to the effects of air pollution, which itself may be an outgrowth of systemic racism related to factors such as residential segregation (Burrows, Luo, Cai, & Aschebrook‐Kilfoy, 2025). However, such research in children and youth remains limited, but is essential given that these exposures are likely to be chronic over time, beginning in childhood and setting the foundation for mental health into adulthood (Reuben et al., 2021).

Finally, consistent with decades of research on risk and resilience in mental health, work on climate‐related environmental stressors will benefit from a more explicit focus on resilience‐promoting factors. These may exist at the level of the individual or at a broader ecological level, such as that of neighbourhoods or communities. What are the promotive or protective effects of green spaces (e.g. parks and forests) and blue spaces (e.g. rivers and lakes) against the effects of climate‐related stressors? How do other enriching factors, such as perceived social support, community cohesion, and availability of heat‐reducing and clear‐air community facilities, directly counter or buffer against heat and air pollution exposure? Understanding the resilience‐promoting effects of these and other factors may inform community‐ and individual‐level interventions and potentially shape policies centred on reducing exposure to harmful climate‐related stressors, while imbuing communities with factors that help to offset certain exposures. In terms of specific practices, improving air‐quality standards, particularly in highly polluted urban centres, may itself yield positive benefits for child outcomes. Environmental risk assessments during the prenatal and early childhood period may help to identify at‐risk groups, though the predictive accuracy of this practice must first be demonstrated. Prioritization of care and support for socioeconomically or environmentally disadvantaged groups may be especially useful in reducing mental health disparities. Moreover, building resilience through community investment will require focused attention on the factors shown to counter, offset, or mitigate the effects of climate‐related stressors. In under‐resourced heat‐stricken areas, availability of reliable water and sanitation may help reduce exposure to heat‐related contaminants, while community initiatives centred on providing cooling tools (fans, canopies, cool surface technologies, etc.) can help to support temperature regulation (Cuartas, Balza, Camacho, and Gómez‐Parra 2026). Likewise, in high pollution areas, provision of indoor air purifiers, use of air quality monitors, and development of public awareness campaigns may prove useful in certain circumstances. The range of possible interventions is reviewed in detail elsewhere (Strong et al., 2025), but is promising in suggesting that the burden of climate‐related environmental stressors may be at least partially overcome through targeted, sustained, and multilevel investment in both individuals and the communities in which they reside.

## Ethical considerations

Ethical approval is not applicable to this Editorial Perspective article.

## Data Availability

Data sharing not applicable to this article as no datasets were generated or analysed during the current study.
